# Reflections on the role of Peer Researchers in eating disorders: identifying challenges, best practices, and future directions

**DOI:** 10.1186/s40337-026-01757-w

**Published:** 2026-10-08

**Authors:** James Downs, Ellen Maloney, Anna Carnegie, Kai S. Thomas, Laura Chapman

**Affiliations:** 1 Independent Researcher, Cardiff, Wales; 2 University of Edinburgh, Edinburgh, Scotland; 3 King’s College London, London, England, UK; 4 School of Psychology, Cardiff University, Cardiff, Wales; 5 Bristol Medical School, University of Bristol, Bristol, England, UK

**Keywords:** Eating disorders, Peer research, Lived experience, Epistemic justice, Co-production, Patient and public involvement, Research ethics, Trauma-informed practice, Participatory research, Methodology

## Abstract

The involvement of people with lived experience has become increasingly prominent across mental health research, with co-production and Peer Researcher roles presented as ways of improving the relevance, ethics, and legitimacy of research. Within eating disorder research, however, the integration of Peer Researchers remains inconsistent and is shaped by a range of structural, cultural, and epistemic barriers. Although a growing literature addresses lived experience involvement in eating disorder research, there has been limited critical examination of how Peer Researcher roles operate within a field marked by stigma, narrow recovery narratives, contested notions of credibility, and constrained resources. This paper offers a critical, experience-informed perspective on Peer Research in eating disorders, drawing on the authors’ lived and professional experience alongside a selective synthesis of scholarship on co-production, lived experience research, and epistemic justice. We identify recurrent challenges in the implementation of Peer Researcher roles, including unequal recruitment and representation, trauma- and stigma-related harms, limited power-sharing, inadequate attention to welfare, uneven training and career development, and the effects of working within a comparatively small and under-resourced field. We argue that meaningful inclusion of Peer Researchers depends not simply on their presence within research teams, but on improving the conditions within which they participate. This includes greater flexibility, shared decision-making, appropriate support, and recognising experiential knowledge as a valuable form of expertise. This paper contributes a framework for understanding these challenges and offers practical considerations for developing ethical, just, and sustainable approaches to Peer Research that will improve the relevance and legitimacy of eating disorder research more broadly.

## Abstract (plain language summary)

People with lived experience of eating disorders are increasingly being involved in research, including through roles as Peer Researchers, where they work as part of research teams rather than only recruited as participants. These approaches are often described as ways of making research more relevant, ethical, and grounded in real-world experience. However, in eating disorder research, Peer Researcher roles are still used inconsistently and can be limited by stigma, narrow ideas about recovery, unequal power relationships, and lack of practical support. In this paper, we draw on our own experiences alongside published research to examine some of the main challenges involved in including Peer Researchers in eating disorder research. These include who gets recruited, how much influence Peer Researchers actually have, how trauma and stigma can affect participation, whether welfare needs are recognised, and what kinds of training, support, and career opportunities are available. We also argue that these issues are shaped by the wider context of eating disorder research as a relatively small and under-resourced field. The paper highlights practical ways of supporting more meaningful involvement and argues that including Peer Researchers well requires cultural and structural change, not just good intentions.

## Introduction

### Co-production and Peer Research in mental health

Across mental health research, involvement of people with lived experience has expanded considerably over the past two decades [1]. A substantial body of literature suggests that meaningful lived experience involvement can broaden research agendas, surface neglected priorities, challenge deficit-based framings, and produce knowledge that is more closely attuned to the realities of care and recovery [1–5]. Co-produced and lived experience-led approaches have been associated with improvements in the relevance, ethical quality, and social legitimacy of research [6–9]. Co-production can also challenge long-standing hierarchies between professional and experiential forms of knowledge, reflecting growing recognition that experiential knowledge is a legitimate and necessary form of expertise within mental health science [1, 3, 9–14].

Within this broader landscape, Peer Researchers are increasingly common roles. Peer Researchers are people with lived and/or living experience who are embedded as members of research teams rather than positioned solely as participants or external advisors [15]. Their roles include shaping research questions, study design, recruitment, data generation, analysis, interpretation, dissemination, and leadership. Peer Research may therefore function as a structural intervention within research systems, redistributing authority by challenging who is considered capable of producing knowledge and whose interpretations are able to influence it [1, 16, 17].

However, co-production literature also documents the risks and ethical concerns that can accompany lived experience involvement when it is poorly resourced or weakly embedded within institutional structures. These include tokenism, stigmatisation, unequal power relations, emotional labour and exploitation, and the tendency for involvement to remain procedural or abstracted from wider social and cultural contexts [1, 3, 12, 13, 18–21]. Such problems may arise through interpersonal attitudes and local research cultures, but they are also shaped by broader systemic forces including funding arrangements, governance requirements, employment structures, and project timelines. These factors help explain how unequal participation of people with lived experience is produced and where responsibility for change lies. They do not, however, render the effects of inequity as inevitable or ethically neutral. Meaningful co-production requires sustained attention to epistemic justice, cultures of mutuality and care, and the wider conditions within which eating disorder research is organised.

### Lived experience in eating disorder research

The growth of lived experience involvement in eating disorder research is reflected in an emerging literature on co-production, participatory methods, and lived experience-informed approaches [22–26]. Recent work has highlighted both the promise of these developments and their current limitations. A scoping review of lived experience co-design in eating disorder research identified 76 studies, but found substantial variation in how lived experience was incorporated, ranging from opportunistic consultation to full integration across the project lifecycle [25]. Methodological reporting was often limited and inconsistent, with many studies stating that lived experience had been included while providing little detail on roles, processes, or actual influence on research decisions [25]. The presence of lived experience within a project therefore reveals little, by itself, about the authority afforded to contributors or the extent to which their knowledge altered the research.

Research has also begun to examine how lived experience collaboration is perceived by different groups involved in eating disorder research. A recent survey of people with lived experience, carers, clinicians, and researchers suggests widespread recognition of the value of lived experience collaboration for producing more meaningful research and improving treatment [26]. Respondents also identified ongoing uncertainties about appropriate support structures, potential risks to contributors, especially around relapse, and the need for clearer guidance on safe and effective collaboration [26]. These concerns require serious engagement, while avoiding assumptions that living experience or fluctuating health automatically disqualifies participation. The possibility of distress places greater importance on collaborative planning, flexibility, and appropriate support, rather than establishing exclusion as the default response.

Lived experience accounts within the eating disorder field consistently document the effects of stigma, diagnostic stereotyping, and contested constructions of credibility and recovery within services and research cultures [21, 27–29]. These dynamics shape how people with eating disorders are perceived as knowers and have been linked to forms of epistemic injustice that affect whose experiences are treated as legitimate within both care and research [29]. People may be viewed as lacking insight, insufficiently motivated, unreliable, or too closely identified with illness, particularly where their accounts challenge dominant clinical understandings of recovery, risk, or treatment.

Constructions of recovery are especially consequential for access to Peer Research roles. Eating disorders may involve sustained recovery, relapse, fluctuation, partial recovery, ongoing illness, or long-term management [28, 30, 31]. Nevertheless, opportunities may favour people who are perceived as sufficiently recovered, emotionally distanced from their experiences, or able to present narratives that conform to established expectations. People with living experience may conversely be judged too vulnerable or clinically risky to contribute. These dynamics can play out within research teams but are often enforced - or reinforced - by institutional structures. Such positions can narrow the range of experiential knowledge entering research.

Eating disorder research is also conducted within a comparatively small and under-resourced field [32–35]. Limited funding, short project cycles, competitive grant processes, and fragmented career pathways affect when Peer Researchers can enter projects, how their labour is remunerated, and whether roles can be sustained. These pressures may lead teams to involve Peer Researchers only after funding has been secured and key decisions have already been made. The resulting difficulties should be understood as institutional and systemic concerns, which also affect early career researchers, rather than treated solely as shortcomings of individual researchers or as fixed limits on what ethical collaboration can require.

### The value of Peer Researchers in eating disorder research

The case for Peer Research in eating disorder research rests on longstanding limitations in how knowledge in this field has been produced. Despite decades of research and some increasing investment in eating disorder science, important gaps remain in both the evidence base and its translation into meaningful care [32, 35]. These gaps reflect wider structural dynamics that prioritise certain populations and forms of knowledge, most often those aligned with white, cisgender, female, thin-bodied, middle-class, and non-disabled norms, while marginalising experiences that fall outside these frames [36–39]. For people with intersecting experiences of marginalisation, including racialised people, neurodivergent people, disabled people, people in larger bodies, LGBTQ+ people, older people, and those with co-occurring trauma, these exclusions have tangible consequences, contributing to delayed diagnosis, misrecognition of symptoms, reduced access to care, and treatment models that may not reflect lived realities [40–45].

The exclusion of lived experience can be seen as both a cause and a consequence of these knowledge gaps. While lived experience involvement is increasingly encouraged by funders and institutions [46, 47], it is often confined to advisory or consultative roles external to the main sites of knowledge production [22, 25, 26]. Historically, people with eating disorders and their caregivers have often been positioned as unreliable narrators of their experiences [28, 29, 48], and their accounts may continue to be discounted as symptoms rather than treated as sources of insight.

Peer Research can address these limitations by bringing experiential knowledge into the sites where research agendas are set, concepts and measures are developed, data are interpreted, and meanings are negotiated. Peer Researchers may identify neglected questions, expose assumptions embedded within study designs, improve the accessibility and acceptability of research procedures, and interpret findings in relation to embodied, relational, and social contexts that may otherwise remain obscured. Whilst experiential knowledge is diverse and situated, and no individual can represent an entire population, its inclusion nevertheless expands the range of perspectives through which research can be examined and challenged.

From this perspective, Peer Research is an epistemic justice intervention. It challenges the unequal distribution of credibility and interpretive authority within research [9, 10, 12, 29]. Epistemic equality does not require differences in training, role, or formal responsibility to disappear, and some research activities require specialist methodological, technical, or regulatory competence. The relevant question is how these differences are organised, and whether they uphold a hierarchy in which academic or professional knowledge routinely overrides experiential expertise, including on matters to which it is directly relevant.

The potential of Peer Research fundamentally depends on the conditions under which Peer Researchers participate. These include the point at which they enter a project, whether, and at what level, their work is remunerated, how roles and decisions are negotiated, whether disagreement can be expressed safely, how accessibility and welfare are supported, and whether experiential contributions can materially alter the direction or interpretation of the research. Without adequate resourcing, institutional commitment, and meaningful influence, Peer Research may be absorbed into existing systems in ways that welcome lived experience rhetorically while leaving research cultures substantively unchanged [1, 16, 18].

### Contribution and aims of this paper

Many of the challenges associated with Peer Research, including tokenism, emotional labour, unequal power, and insecure employment, have been discussed across mental health and participatory research [1, 3, 17, 21]. Their expression within eating disorder research is shaped by particular constructions of recovery, vulnerability, credibility, severity, and expertise, which play out within a highly pressurised and unevenly resourced research ecosystem [25, 26, 28, 29, 32, 35]. The interaction between these conditions has received less sustained attention.

This paper offers a critical, experience-informed examination of Peer Researcher roles in eating disorder research. Drawing on the authors’ lived and professional experiences alongside a selective and interpretive synthesis of scholarship on co-production, lived experience research, and epistemic justice, we examine how unequal participation may be produced through ordinary research practices and institutional arrangements, including when individual researchers are committed to lived experience involvement.

Specifically, we aim to:

-  situate eating disorder Peer Research within the wider lived experience research landscape and identify the cultural, clinical, epistemic, and structural conditions that shape these roles;
-  examine recurrent challenges affecting recruitment and representation, trauma, stigma and welfare, power-sharing and decision-making, training and career development, and resourcing;
-  consider how experiential, methodological, and professional forms of expertise can be brought into dialogue without reproducing a general hierarchy of knowledge; and.
-  identify practical and structural directions for developing more ethical, just, and sustainable approaches to Peer Research in eating disorders.

## Methodological approach and positionality

### Design and methodological orientation

This paper is positioned as an experience-informed critical interpretive commentary. It is not a systematic review and does not claim exhaustive identification or formal aggregation of all relevant evidence. Rather, it combines published empirical, theoretical, and experiential scholarship with collaborative reflection among authors who work as Peer and lived experience researchers in different ways. The purpose is interpretive and synthesising: to develop a situated account of how cultural, epistemic, ethical, and structural conditions shape Peer Researcher roles in eating disorder research, and to formulate an argument that can inform practice and institutional change.

Literature engagement was informed by the activities associated with critical interpretive synthesis (CIS): formulation of an open question; iterative searching; purposive source selection; critical appraisal; extraction and interpretation of concepts; and development of a synthesising argument [49]. Rather than claiming a full formal CIS, we describe our approach as ‘informed by’ the principles of CIS, because the work has been developed through an iterative process rather than a prospectively registered review protocol. As such, the paper was not designed to provide exhaustive coverage of all published literature.

### Research question, searching, and source selection

The open question guiding the paper was: how do the cultural, epistemic, ethical, and structural conditions of eating disorder research shape the possibilities, risks, and implementation of Peer Researcher roles? Literature was identified iteratively during manuscript development through the authors’ existing knowledge of eating disorder and co-production scholarship, targeted searches of relevant academic databases and search engines, and backward and forward citation searching from conceptually important sources. Search concepts included combinations of “peer researcher”, “lived experience researcher”, “co-production”, “co-design”, “participatory research”, “eating disorder”, “mental health”, “epistemic injustice”, “trauma-informed”, “emotional labour”, “power sharing”, “research ethics”, “training”, “career progression”, “funding”, and “governance”.

Sources were selected purposively for their ability to illuminate, extend, or challenge the developing analysis rather than according to methodological design alone. We included empirical studies, reviews, methodological papers, conceptual scholarship, commentaries, and lived experience-led work relevant to Peer Research, co-production, epistemic justice, eating disorder research cultures, welfare, and structural conditions. Eating disorder-specific evidence was prioritised where available, while broader mental health and participatory research literature was used where it addressed questions not yet developed within the eating disorder field. Sources were not excluded solely because they offered critical, experiential, or non-traditional forms of knowledge.

### Critical appraisal and synthesis

Quality appraisal was integrated into the interpretive process rather than undertaken through a standardised scoring tool. Sources were considered in relation to the transparency of their methods or argument, the adequacy of the evidence presented, their conceptual contribution, and the relevance of their findings to Peer Research. Particular care was taken when drawing on literature from broader mental health or participatory research, recognising that its findings could not be assumed to transfer directly to eating disorder research settings.

The analysis was developed through sustained, iterative dialogue between authors. JD led the initial literature engagement and wrote the first draft. JD, EM, and AC met regularly to reflect critically on their roles, compare the literature with their experiences across different Peer Research contexts, and identify recurring patterns and points of divergence. KT contributed additional critical analysis, including perspectives arising from research with trans, gender-diverse, and neurodivergent communities. LC reviewed and contributed to later drafts.

Interpretation developed through repeated movement between the literature, the authors’ experiences, and successive versions of the manuscript. Emerging claims were examined for the extent to which they arose from published evidence, situated experience, or an interaction between the two. Differences between authors were treated as analytically productive, particularly where they revealed contrasting understandings of vulnerability, credibility, professional responsibility, methodological expertise, and power.

This process generated five connected domains: recruitment and representation; trauma, stigma, and welfare; power-sharing and decision-making; training and career development; and structural resourcing. It also produced the synthesising argument that meaningful Peer Research depends on the conditions under which experiential expertise enters, influences, and is sustained within research, and on how responsibility for those conditions is distributed across projects, institutions, funders, and governance systems, not just individual researchers themselves.

### Epistemological stance and collective reflexivity

Empirical, theoretical, and experiential sources were held in dialogue throughout the paper. This does not mean that all claims are treated as identical or exempt from scrutiny. Rather, it reflects a pluralistic stance in which the credibility of knowledge is not determined in advance by whether it originates in personal experience, professional practice, or conventional research [14, 29]. Personal experience was used as an analytic resource through which institutional practices, ethical tensions, and epistemic assumptions could be examined, while being interpreted alongside and sometimes challenged by published scholarship and the differing experiences of co-authors.

Knowledge development was treated as situated and co-constituted, emerging through interaction, negotiation of difference, and reflexive engagement [2, 20, 29, 50]. Our collective reflexivity involved attention not only to interpersonal relationships within the author group, but also to the structural conditions through which some forms of lived experience become more readily legible within academia than others [1, 12, 21]. Lived experience research can privilege whiteness, middle-classness, professional fluency, formal education, stability, and proximity to established services or research networks while marginalising those whose experiences or communication do not fit these expectations [51–54]. We therefore present this paper as a situated contribution rather than a representative account.

### Individual positionalities

JD is an independent Peer Researcher working across universities, professional bodies, and research projects. His perspective is shaped by longstanding experience of eating disorders, mental distress, physical disability, and neurodivergence, alongside training and professional experience in psychological therapy and mental health research. These experiences orient his analysis towards embodied knowledge, epistemic pluralism, and the effects of research and clinical systems on people with complex or non-linear trajectories.

EM is employed as a Peer Researcher in a role where lived and living experience of autism and an eating disorder is explicitly recognised as expertise. Her perspective brings particular attention to accessibility, fluctuating needs, appropriate support, and the importance of Peer Researchers participating as core members of research teams.

AC works in a research role that was not originally designated as Peer Research, while actively drawing on lived experience of an eating disorder in the coordination of a large-scale project concerning eating disorder service data. Her perspective is informed by navigating overlapping professional and experiential identities and contributes attention to role clarity, organisational preparedness, and the demands placed on people who bring lived experience into established research environments and roles.

KT is a postdoctoral researcher whose work focuses on eating disorders among trans, gender-diverse, and neurodivergent people. His research is informed by lived experience as a trans person who has experienced an eating disorder. His perspective contributes attention to intersectionality, the exclusion of minoritised populations, and the development of opportunities for lived experience leadership.

LC is a postdoctoral researcher whose lived experience of an eating disorder contributed to her decision to pursue research in this field. Her qualitative work examines the experiences of people affected by eating disorders and seeks to inform prevention and treatment. Her position contributes attention to the relationship between experiential and methodological expertise and to the ways lived experience can shape research in the context of an academic role.

## Challenges and suggested practices for including Peer Researchers in eating disorder research

Through critical discussion of our experiences in relation to the existing literature, we identified five connected areas in which challenges commonly arise in the implementation of Peer Researcher roles in eating disorder research. These concern recruitment and representation; trauma, stigma, and welfare; power-sharing and decision-making; training, development, and career progression; and structural resourcing. These areas often overlap in practice: recruitment is shaped by who can afford to participate, welfare by how roles and workloads are organised, power-sharing by when and how Peer Researchers enter a project, and career progression by whether institutions invest in sustained rather than episodic involvement. In each section, we describe the challenge and consider practices that may support more ethical and meaningful participation.

### Recruitment and representation

#### Challenges: unequal access and narrow constructions of expertise

A persistent challenge in integrating Peer Researchers into eating disorder research is the lack of diversity in who is recruited and supported to take up these roles. Opportunities often favour individuals who meet conventional definitions of recovery, are already connected to research networks or services, or hold formal diagnoses [28, 30, 31]. Recruitment processes frequently rely on self-selection, which may privilege those with stable health, financial security, confidence in professional environments, or established support networks [3, 21, 55]. This may exclude people with valuable insights who are distant from services, lack access to diagnosis or treatment, do not meet criteria suggesting that they are sufficiently “recovered”, or mistrust healthcare and academic systems because of previous trauma or structural exclusion [42, 56, 57].

These dynamics are compounded for people facing intersecting marginalisations related to race, gender, disability, neurodivergence, age, and socioeconomic position [40, 42, 44, 45]. LGBTQ+ people, particularly trans and non-binary people, men, disabled people, neurodivergent people, people in larger bodies, and older people may be excluded when their experiences do not align with dominant stereotypes of eating disorders [39, 44, 58–60]. Racialised communities may encounter diagnostic overshadowing, cultural incompetence, and reduced access to treatment, which in turn reduces proximity to research opportunities [41, 56, 61, 62]. These inequalities can be reinforced within academia, where structural racism, classed expectations, and unequal access to research careers continue to shape who is able to enter and remain in research roles [53, 54].

Recruitment processes may also (re)produce representational burdens. Peer Researchers can be expected to speak for an entire population or identity group, particularly where only one person with lived experience is included. This can flatten internal differences within communities and place disproportionate responsibility on individuals to provide a definitive “lived experience perspective”, when experiential expertise is situated and plural by its nature.

Without deliberate efforts to address these patterns, the diversity and depth of lived experience represented in eating disorder research will remain constrained, with consequences for the relevance and inclusivity of the knowledge produced.

#### Suggested practices: widening access and clarifying representation

Across mental health and eating disorder research, a growing body of work has begun to address these recruitment challenges by broadening access to lived experience participation [24–26, 63]. Rather than relying solely on established research or clinical networks, more inclusive projects have used recruitment strategies that intentionally seek participation from communities historically underrepresented within eating disorder research.

Participatory work involving Autistic people with eating disorders illustrates how adapting recruitment processes and research design can improve accessibility for neurodivergent contributors [64–66]. Research with and led by transgender and gender-diverse people similarly highlights the importance of recruiting beyond specialist eating disorder services, since many people encounter barriers to diagnosis and treatment that would otherwise exclude them from research participation [58, 67, 68]. Community-based approaches have been used to include people who are geographically or socially distant from specialist services, including Indigenous communities whose experiences may be marginalised within dominant clinical frameworks [69]. Innovative qualitative methods, including the use of objects, have also been used to explore eating disorder recovery among people from marginalised communities [70].

An important feature of these examples is their broader understanding of experiential expertise, extending beyond people who have completed treatment or meet formal diagnostic criteria to include those whose experiences fall outside conventional clinical or research pathways. In our collective experience, involving lived experience collaborators in the design of recruitment materials, role descriptions, and selection processes can make opportunities more accessible to people unfamiliar with academic environments.

Recruitment processes should also be explicit about what kind of contribution is being sought. A role may require insight into a particular experience, community, method, or aspect of research, but these requirements should be justified rather than inferred from narrow ideas about diagnosis or recovery. Selection criteria should distinguish between what is genuinely necessary for the work and what simply reflects familiarity with conventional academic practice.

Where a project seeks insight relevant to diverse populations, this may require more than recruiting a single Peer Researcher. Funding structures should support consideration of multiple contributors, partnerships with community organisations, embedded advisory structures, and continuing mechanisms of accountability to the communities concerned. Research teams can also avoid presenting an individual contributor as representative of a wider group unless that role has been explicitly negotiated and supported.

These practices cannot by themselves overcome the wider inequalities that shape who is able to participate, whose experience is recognised as relevant, and whose knowledge is treated as credible. Recruitment is therefore an early site at which inclusion, legitimacy, and the boundaries of recognised expertise are actively shaped.

### Trauma, stigma, and welfare

#### Challenges: emotional labour, stigmatisation, and fluctuating capacity

Peer Researchers in eating disorder research may encounter distress through exposure to difficult material and through the wider discourses, assumptions, and practices that continue to shape eating disorder care and research. Lived experience literature consistently shows that people with eating disorders may experience clinicians and services as stigmatising, disbelieving, or morally judgemental, with recurring themes including being seen as manipulative, difficult, attention-seeking, or insufficiently motivated to recover [27, 29, 71, 72]. These experiences may reproduce epistemic injustice by shaping whose accounts are treated as credible, whose interpretations are taken seriously, and which kinds of experience are recognised as authoritative [29].

In eating disorder contexts, this may be intensified by diagnostic and treatment cultures that frame ambivalence, disagreement, or alternative understandings of recovery as evidence of pathology rather than insight [28, 30, 71]. For Peer Researchers, this can mean that the perspectives for which they are invited into research remain subtly mistrusted, reframed, or contained within it.

The emotional consequences of this are increasingly recognised. In the wider co-production literature, emotional labour is understood as central to lived experience involvement rather than incidental to it [1, 3, 18, 21]. Within eating disorder research, this labour may include reading, hearing, or analysing accounts of illness, treatment, stigma, and recovery in ways that resonate with, unsettle, or conflict with one’s own history. Survey research on lived experience collaboration in eating disorder research has also suggested differences between the risks prioritised by researchers and those identified by people with lived experience, particularly in relation to stigma and relapse [26]. This raises the possibility that some burdens associated with participation may remain insufficiently recognised within research teams.

Eating disorders are often marked by fluctuation, partial recovery, relapse, relative stability despite illness, and long-term management rather than a simple transition from illness to wellness [28, 30, 31]. Yet research environments may still operate with implicit assumptions that contributors are either fully recovered and therefore unaffected by the subject matter, or currently too unwell for involvement to be appropriate. This leaves little room for those whose experiences fall between these poles.

The content, organisation, and culture of research work may intensify these pressures. Topics such as eating practices, body image, service use, weight stigma, coercion, and treatment experiences can be emotionally demanding, while meetings arranged around mealtimes, rigid deadlines, expectations of uninterrupted productivity, or limited scope to step back from particular tasks may sit uneasily alongside fluctuating health needs. Stigma may also shape whether it feels possible to disclose such needs, as requests for flexibility can carry fears of being seen as difficult, insufficiently well, or unprofessional.

Concerns about welfare may also lead to protective exclusion. Researchers and institutions hold genuine duties of care and may be accountable for the safety of staff and collaborators. However, these responsibilities can become paternalistic where decisions about readiness, risk, or appropriate participation are made without transparent and collaborative discussion with people with lived experience themselves. Blanket assumptions based on diagnosis, relapse history, or current treatment status may reproduce the same judgements about competence and credibility that Peer Research is intended to challenge.

#### Suggested practices: collaborative and proportionate support

The most helpful approaches identified in mental health and eating disorder literature are those that treat trauma, stigma, emotional labour, and fluctuating capacity as foreseeable aspects of participatory work [18, 19]. Trauma-informed participatory research offers a useful framework, organised around anticipation, flexibility, reflection, safety, and relational trust [73, 74]. In practice, this may include discussing possible risks in advance, building in space for debrief and reflection, recognising that participation may vary over time, and attending to the conditions that allow people to contribute without concealing difficulty [73, 74].

Within eating disorder research, these principles are beginning to be adapted in more field-specific ways. Research on ethical co-production with Autistic people with eating disorders has highlighted the importance of anticipating access needs early, adapting formats of participation, allowing people to opt in or out of particular tasks, and maintaining ongoing dialogue rather than assuming uniform or stable capacity [66]. These adaptations can be understood as part of good research design rather than as exceptional concessions.

Practically, this may include attention to when meetings are scheduled, scope to step back from particular tasks or materials, choice over forms of participation, and recognition that topics such as body image, weight stigma, coercive treatment, or recovery narratives may affect contributors differently. In our experience, modest adjustments can have substantial effects: flexibility in scheduling, openness to different working rhythms, clarity that stepping back from a task will not be interpreted as failure, and explicit recognition that needs may change over the course of a project.

Decision-making about which activities to be involved with and the support needed must be shared rather than presumed. Peer Researchers should be able to identify what forms of support are helpful, what information they wish to disclose, and how concerns should be addressed if circumstances change. These discussions should also clarify the boundaries between research supervision, occupational support, peer support, and clinical care. Research teams should not assume a therapeutic role for which they are neither trained nor responsible, but neither should emotional labour be treated as a private matter for individuals to manage alone.

Risk should be considered in relation to specific tasks and contexts rather than attached to lived experience or particular diagnoses as general categories. Participation in a sensitive interview, analysis of distressing material, public disclosure, and involvement in statistical work may carry different forms of risk for different people, at different times, and may require different support. Proportionate, role-specific planning allows welfare and inclusion to be considered together. Importantly, flexible and trauma-informed practice requires time, skill, and resources. Its implementation should therefore be recognised within project planning and budgets rather than assumed to arise through the goodwill or unpaid emotional labour of individual team members.

Anti-stigma practice is also important here, and should draw attention to the everyday language through which eating disorders, treatment, recovery, severity, and risk are discussed in research contexts [71]. This includes being open to people with lived experience questioning and reshaping terms that feel reductive, moralising, exclusionary, or overly certain, and recognising that concepts treated as routine within clinical and research cultures may carry very different meanings for those who have lived through them [71, 75–77].

### Power-sharing, expertise, and decision-making

#### Challenges: limited influence and unequal authority

A recurring challenge in Peer Research is that lived experience may be included without being given meaningful influence over the research itself. Peer Researchers may be brought in after key decisions have already been made, invited to comment on plans rather than shape them, or included in ways that leave core authority over design, analysis, interpretation, and dissemination elsewhere. Across the co-production literature, this problem is well recognised: involvement may be present and visible while remaining tightly bound in practice [1, 13, 17].

In our own experiences, these dynamics are often less overt than simple exclusion. Lived experience may be welcomed mainly in forms that fit established project structures, such as personal reflection, illustrative testimony, or validation of decisions shaped elsewhere. Peer Researchers may be invited into the room but not into the parts of the process where concepts are defined, assumptions are challenged, or interpretations are negotiated. This aligns with findings from eating disorder research showing substantial variation in the extent and influence of lived experience involvement and limited reporting of how far such involvement affects decision-making [25].

Concerns about vulnerability, relapse, readiness, or boundaries may also steer Peer Researchers away from aspects of the work considered too sensitive, technical, or consequential. While these concerns may be well-intentioned, they can narrow lived experience roles and preserve existing distributions of authority. Neill et al. identified continuing uncertainty around risk, support, and the circumstances in which lived experience collaboration is considered appropriate [26].

Questions of power-sharing also intersect with methodological expertise and formal accountability. Some research activities require advanced technical training, and chief investigators, sponsors, or employing institutions may retain legal and professional responsibility for particular decisions. These realities are relevant to how collaboration is organised. They do not establish that all consequential decisions should remain within conventional professional hierarchies. Technical competence in one domain does not confer exclusive authority over the framing, interpretation, ethical meaning, or lived relevance of the research.

#### Suggested practices: differentiated expertise and meaningful influence

More meaningful power-sharing depends on practical structures. These may include involving Peer Researchers from the outset of a project; agreeing how decisions will be made; being transparent about roles, authorship, payment, and accountability; and ensuring that people with lived experience are included in analysis, interpretation, and dissemination rather than only in advisory or consultative capacities [1, 3, 8].

Clearer differentiation between forms of expertise can strengthen rather than weaken collaboration. Statistical modelling, trial design, data governance, qualitative interpretation, experiential understanding, community knowledge, and ethical judgement each involve distinct forms of competence. Teams should identify where specialist training is required, where knowledge can be developed through support and mentoring, and where decisions benefit from the interaction of several forms of expertise.

Formal responsibility should also be distinguished from exclusive decision-making authority. A chief investigator may remain accountable for regulatory compliance, while still sharing the development of research priorities, interpretation of findings, and decisions about language, accessibility, or dissemination. Where a decision cannot be shared because of legal, technical, or governance requirements, the reasons should be made explicit and open to scrutiny.

Practical steps may include:

- agreeing decision-making processes at the beginning of a project;
- identifying which decisions will be shared, delegated, or subject to formal accountability;
- ensuring that Peer Researchers receive the information and training required to participate meaningfully;
- creating routes for disagreement and challenge;
- documenting how lived experience input has influenced the project; and.
- reviewing these arrangements as the work develops.

These practices are consistent with calls by Downs et al. (2025) for a more "dialectical" approach to the co-production of knowledge in eating disorder research, which includes practices such as consultation with the other, phenomenological empathy, valuing dissonance as well as consonance, observing limits, and recognising fallibility [29].

Perhaps the clearest marker of genuine collaboration is whether lived experience input can change the work itself. Examples from eating disorder research have shown how lived experience can reshape understandings of service accessibility, recovery, chronicity, and treatment goals, rather than simply being added as contextual testimony [28, 76, 78].

Meaningful influence does not require every contributor to participate in every task or every decision, either. Rather, people with lived experience require access to the parts of the research where their expertise is relevant, with sufficient support to contribute, and assurance that disagreement will be considered rather than dismissed through appeals to status or professional authority.

Further work is needed to evaluate how power-sharing is enacted and with what effects. The field would benefit from more transparent reporting of whether lived experience involvement has substantively influenced research questions, methods, interpretations, and decisions, rather than remaining visible only at the level of authorship statements or acknowledgements [3, 8, 17, 25, 63].

### Training, development, and career progression

#### Challenges: uneven preparation and insecure career pathways

A recurring challenge across participatory mental health research concerns the limited availability of structured training pathways and sustainable career progression for people with lived experience working within research teams, alongside limited preparation of wider research teams to work in genuinely co-produced ways [3, 79]. Although funders and institutions increasingly encourage lived experience involvement [46, 47, 80], this expansion has often taken the form of short-term project roles rather than longer-term research positions. Many Peer Researchers therefore remain employed on fixed-term or consultancy arrangements tied to individual studies, limiting opportunities to develop sustained expertise or academic careers [3, 79].

Training within projects may focus narrowly on enabling lived experience contributors to support pre-existing research designs, for example through interviewing or coding, rather than equipping all members of the team to share power, work reflexively across different forms of expertise, and involve Peer Researchers in shaping research questions, methodologies, interpretation, and theory [14]. People with lived experience may accumulate substantial informal expertise without clear routes for translating it into recognised research careers, while researchers without lived experience may receive little preparation in the relational, methodological, and ethical demands of co-production [1, 79].

Peer Researchers may also encounter an unequal expectation to acquire conventional academic skills while the wider team is not expected to develop competence in co-production, accessibility, reflexivity, or the ethical use of experiential knowledge. This positions professionalised research practice as the norm to which Peer Researchers must adapt and may leave existing research cultures comparatively unchanged [1].

Conversely, formal training and methodological development remain important. Peer Researchers should not be restricted to contributing personal testimony or assumed to possess research competence solely by virtue of lived experience. Access to training can widen the roles available to them and support progression into analysis, design, grant development, leadership, and methodological innovation. The issue is therefore how training is offered, valued, and connected to genuine opportunities.

#### Suggested practices: reciprocal learning, recognition, and progression

Recent literature points to initiatives designed to strengthen training and progression for lived experience researchers. These include formal programmes in qualitative methods, participatory design, analysis, and grant development, alongside mentorship and supervision structures that recognise the challenges of working across academic and experiential knowledge systems [8, 63, 79].

Equally important is training for researchers, clinicians, and other team members without lived experience in how to work collaboratively and reflexively, including how to share decision-making, respond constructively to challenge, recognise experiential knowledge as expertise, and avoid reducing Peer Researchers to sources of personal disclosure [81]. In our own work, we have found it beneficial for people with lived experience to play a direct role in delivering such training, particularly where this creates space to reflect on power, assumptions, and the practical demands of collaboration.

Training should be reciprocal but not symmetrical. Peer Researchers may need access to methodological and institutional knowledge, while established researchers may need to develop skills in accessibility, relational practice, power-sharing, and epistemic reflexivity. Both forms of development should be treated as core research competencies rather than optional enhancement.

Recognition and remuneration are equally important. Sustainable Peer Research roles require clear employment structures, fair compensation, appropriate authorship and attribution, and opportunities for progression beyond single projects [1, 3, 80]. Career development should include routes into substantive research posts, fellowships, leadership, and principal investigator roles where individuals wish to pursue them. It should also recognise that some Peer Researchers may prefer portfolio, community-based, advisory, or non-academic forms of work. Progression should therefore be understood broadly rather than measured only through conventional academic promotion.

In our experience, structural support can enable Peer Researchers to take on increasingly substantial roles in shaping and leading eating disorder research. Investment in their development has the potential to expand both participation and the intellectual scope of the field [1, 14, 24, 25, 29].

## Structural conditions and responsibility for change

### A resource-limited research ecosystem

The challenges described across the preceding sections are shaped by the broader context of eating disorder research as a comparatively small and under-resourced field. Eating disorder research has been described as operating within a constrained and fragmented ecosystem, in which limited funding, dispersed expertise, and relatively small research networks restrict methodological innovation, workforce development, and sustained collaboration [33, 35]. Compared with many other areas of mental health research, investment remains disproportionately low relative to disease burden and mortality [35]. The field is also often positioned as relatively niche, with a limited number of centres and specialised programmes shaping the production of knowledge [33, 34].

These conditions affect the kinds of participatory and co-produced practices that can be developed and sustained. Participatory research is relational and iterative, requiring time for dialogue, flexibility in design, and continued collaboration across different forms of expertise. These requirements can sit uneasily alongside funding structures organised around tightly specified methods, short timelines, fixed deliverables, and rapid outputs.

The effects are visible across the challenges discussed here. Recruitment may default to existing networks, emotional support may be limited, and opportunities for training, progression, and continuity may remain constrained where lived experience roles are tied to individual studies rather than embedded within longer-term programmes of work.

These conditions also shape the point at which Peer Researchers can become involved. Grant applications often require research questions, methods, budgets, and timelines to be specified before funding is available to employ or remunerate lived experience collaborators. Researchers may therefore face a genuine ethical dilemma: involving people in substantial unpaid pre-award work, or delaying involvement until funding has been secured and important decisions have already been made.

This dilemma should not be interpreted as evidence that meaningful early involvement is inherently unrealistic. Rather, it suggests that current funding and institutional arrangements limit collaboration at the stage when it can have greatest influence. Potential solutions to this challenge include pre-award involvement funds, institutional seed funding, funding for standalone lived experience research groups, development grants, and funding mechanisms that allow project designs to evolve at all stages of the lifecycle.

### Ethics, governance, and institutional systems

Ethics and governance frameworks may also shape Peer Research roles in ways that are insufficiently examined. Our range of personal experiences show how Peer Researchers can occupy an ambiguous position between employee, collaborator, advisor, and person with lived experience. Governance processes developed primarily for research participants may be applied to them in ways that overemphasise vulnerability or restrict access to potentially sensitive material, while employment systems may fail to recognise the support and flexibility required in experiential roles.

Ethical review has an important role in protecting people from avoidable harm. However, protective requirements should be proportionate to the role and should not assume that disclosure of lived experience creates incapacity. Ethics committees, sponsors, and institutions should distinguish clearly between research participants and research collaborators, assess risk in relation to specific activities, and consider the harms of exclusion alongside the harms of participation.

Institutional systems also affect payment, contracts, access to occupational support, data access, training, and progression. Delays in payment, restrictions created by welfare benefits, consultancy arrangements without employment protections, and short-term contracts can all undermine participation. These are not administrative details peripheral to co-production; they directly shape who can afford to contribute and whether involvement is sustainable.

### Shared responsibility

The uneven implementation of meaningful lived experience collaboration reflects both project-level practices and the wider organisation of research structures. Responsibility should therefore be distributed across several levels. Firstly, research teams are responsible for role clarity, accessibility, transparent decision-making, proportionate support, and accurate reporting of the extent of involvement. They should avoid describing work as co-produced where Peer Researchers had little influence over its questions, methods, or interpretation.

More broadly, the institutions within which research teams are situated are responsible for employment routes, payment systems, supervision, training, welfare procedures, and career pathways that make sustained Peer Research possible. Funders are responsible for resourcing involvement before and during projects, allowing realistic timelines, and assessing the quality of participatory practice rather than simply requiring its presence. Ethics and governance bodies are responsible for proportionate review processes that recognise Peer Researchers as research collaborators and avoid reproducing blanket assumptions about vulnerability. The wider eating disorder field is responsible for building shared infrastructure, widening participation, evaluating practice, and developing stronger standards of reporting.

It is important to recognise that individual researchers cannot independently resolve every structural problem. However, it is also important to resist explaining away all the challenges of coproduced research as structural features. Even within limiting systems, individual researchers can identify constraints transparently, advocate for change, and resist passing the existing limitations of research practices onto Peer Researchers through unpaid labour, restricted roles, or insecure participation.

## Conclusion: future directions and implementing change

Peer Researcher roles in eating disorder research form part of a broader effort to reshape how knowledge is produced, whose expertise is recognised, and which questions become available for investigation. The existing literature, together with our experiences, suggests that the value of Peer Research is substantial but unevenly realised. Without deliberate attention to recruitment, representation, stigma, welfare, power, training, and resourcing, lived experience may be included in ways that remain partial, precarious, or tokenistic.

The distinctive conditions of eating disorder research make these questions especially important. Contested ideas about recovery, credibility, vulnerability, severity, and insight can shape who is considered suitable to contribute and how their knowledge is received. A small and under-resourced research ecosystem further affects when Peer Researchers can enter projects, the roles available to them, and whether involvement can be sustained.

Meaningful Peer Research requires attention to both project practice and the systems within which projects operate. Research teams can create clearer roles, support accessibility, recognise emotional labour, involve Peer Researchers in consequential decisions, and make transparent how lived experience has changed the work. Institutions, funders, and governance bodies must provide the employment structures, pre-award resources, training, ethical guidance, and longer-term investment required to make these practices sustainable.

Differences in expertise and formal responsibility will remain within research teams. Ethical collaboration depends on ensuring that these differences are relevant to the decisions at hand and do not become a general hierarchy through which experiential knowledge is routinely subordinated. Methodological competence, professional accountability, and lived experience expertise can strengthen one another when their respective contributions are explicit and open to dialogue. Similarly, welfare and inclusion should be addressed through collaborative and proportionate planning. The possibility of distress or fluctuating health requires responsive support, flexibility, and clear boundaries. It should not become a general basis for excluding people with living experience from influential roles.

The field already contains substantial conceptual and practical guidance for improving Peer Research. Further progress will depend on evaluating how involvement operates in practice, reporting both its scope and its influence, and identifying which changes can be made within projects and which require structural reform. Particular priorities include broader and more equitable recruitment, paid involvement during project development, reciprocal training, sustainable career pathways, proportionate governance, and clearer mechanisms for documenting the impact of experiential knowledge on research.

We hope this paper contributes to a broader shift in the culture of eating disorder research. Peer Researchers should be recognised as legitimate and consequential contributors to knowledge, with the opportunity to influence research questions, methods, interpretations, and outputs. Creating these conditions will require critical reflection from research teams and material change from the institutions and systems that organise research. Such change has the potential to strengthen the quality, relevance, and ethical integrity of eating disorder research itself.

## Data Availability

No datasets were generated or analysed during the current study.
